# Bibliometric analysis of PTEN in neurodevelopment and neurodegeneration

**DOI:** 10.3389/fnagi.2024.1390324

**Published:** 2024-03-22

**Authors:** Yun Zhang, Ya-ting Tan, Mei-juan Wang, Lan Li, Ju-fang Huang, Shu-chao Wang

**Affiliations:** ^1^Department of Anesthesiology, The Second Xiangya Hospital, Central South University, Changsha, Hunan, China; ^2^Department of Anatomy and Neurobiology, School of Basic Medical Sciences, Central South University, Changsha, Hunan, China; ^3^Medical Imaging Center, Qingdao West Coast New District People's Hospital, Qingdao, Shandong, China; ^4^Department of Pathology, The Second Xiangya Hospital, Central South University, Changsha, Hunan, China; ^5^Center for Medical Research, The Second Xiangya Hospital, Central South University, Changsha, Hunan, China

**Keywords:** bibliometrics, CiteSpace, VOSviewer, PTEN, PI3K, neuron, nervous system

## Abstract

Phosphatase and tensin homologue deleted on chromosome ten (PTEN) was initially recognized as a significant regulator of cancer suppression and could impede cancer cell survival, proliferation, and energy metabolism. PTEN is highly expressed in neurons and performs crucial functions in neurogenesis, synaptogenesis, and neuronal survival. Disruption of PTEN activity may also result in abnormal neuronal function and is associated with various neurological disorders, including stroke, seizures, and autism. Although several studies have shown that PTEN is involved in the development and degenerative processes of the nervous system, there is still a lack of in-depth studies that summarize and analyse patterns of cooperation between authors, institutions, countries, and journals, as well as research hotspots and trends in this important field. To identify and further visualize the cooperation and comprehend the development and trends of PTEN in the nervous system, especially in neural development and neurological diseases, we used a bibliometric analysis to identify relevant publications on this topic. We first found that the number of publications displayed a growing trend with time, but this was not stable. Universities, institutions, and authors from the United States are leading in this area of research. In addition, many cutting-edge research results have been discovered, such as key regulatory molecules and cellular mechanisms of PTEN in the nervous system, which may provide novel intervention targets and precise therapeutic strategies for related pathological injuries and diseases. Finally, the literature published within the last 5 years is discussed to identify future research trends regarding PTEN in the nervous system. Taken together, our findings, analysed using bibliometrics, may reflect research hotspots and trends, providing a reference for studying PTEN in the nervous system, especially in neural development and neurological diseases. These findings can assist new researchers in developing their research interests and gaining basic information. Moreover, our findings also may provide precise clinical guidelines and strategies for treating nervous system injuries and diseases caused by PTEN dysfunction.

## 1 Introduction

The phosphatase and tensin homologue deleted on chromosome ten (PTEN) was initially recognized as a significant regulator of cancer suppression by inhibiting the activation of the phosphoinositide 3-kinase (PI3K) molecular pathway, which can impede tumour cell survival, proliferation, and energy metabolism (Luo et al., 2021; Xu J et al., 2021; Blanco et al., 2022). PTEN contains a phosphatase domain at the N-terminus and a C2 domain that binds to the plasma membrane (Campbell et al., 2003). PTEN is both a protein and lipid phosphatase that dephosphorylates serine, tyrosine, and threonine residues on several protein substrates (Hopkins et al., 2018; Hu et al., 2019). Because of PTEN’s phosphatase-dependent function of PTEN, the PI3K-AKT pathway is potently inhibited and disturbs various cellular processes, such as DNA replication, cycle progression, and proliferation (Lunardi et al., 2015; Mathur et al., 2017; Ban et al., 2024). Over the past two decades, researchers have revealed that PTEN plays an essential role both in the normal development of organisms and tumorigenesis (Knobbe et al., 2008; Christine et al., 2022). PTEN has also been found to be highly expressed in neurons and performs crucial functions in neurogenesis, synaptogenesis, and neuronal survival (Wong et al., 2018; Wang et al., 2019; Spina Nagy et al., 2021). Disruption of PTEN activity may also result in abnormal neuronal function and is linked to various neurological disorders, including strokes, seizures, and autism (Chen et al., 2019; Cullen et al., 2023; Fan et al., 2023). For example, a comprehensive library of pathology-associated PTEN mutations was generated by collecting exome and genome sequences from patients with autism, confirming its role as a risk factor for nervous system diseases (Wu et al., 2020). Furthermore, suppressing PTEN is widely regarded as a compelling approach to enhance axon regeneration in impaired or deteriorating neuronal axons (Lu et al., 2014; Nieuwenhuis and Eva, 2022). In accordance with accumulating evidence that PTEN is widely involved in the development and degenerative processes of the nervous system, developing a deeper understanding of the hotspots and trends of PTEN research, especially in neural development and pathological diseases, would assist researchers in understanding the most fundamental, novel, and influential research results, as well as helping new researchers to select potential and valuable research directions.

Review and bibliometrics are two types of study that are useful for summarising and analysing literature in a specific research area (Hu et al., 2022; Zhu et al., 2022; Liu et al., 2023). Compared with reviews, bibliometric analyses, which use mathematical and statistical methods, can evaluate publication quality, citation burst, and scholarly impact, thereby reflecting the current state of related research information (Chen et al., 2020; Shah et al., 2021; Creager et al., 2022; Zhang JH et al., 2023). This discipline can also provide new researchers who aim to enter the field of research with the most fundamental and influential research studies (Yan et al., 2022; Zhang et al., 2022). CiteSpace and VOSviewer are two popular visual software packages for analysis that can help bibliometric researchers arrange large quantities of disordered data into an objective and rigorous network diagram (Yan et al., 2021; Wang and Maniruzzaman, 2022; Zheng et al., 2022). Based on these bibliometric sets, the research results may answer the following questions: What is the pattern of cooperation among authors, institutions, countries, and journals (Halepoto et al., 2022; Pan et al., 2022; Melo et al., 2023)? What is the most influential research topic in a specific field (Halepoto et al., 2022; Pan et al., 2022; Melo et al., 2023)? What are the current research hotspots in the published literature? What might be the future research trends in this specific field (Halepoto et al., 2022; Pan et al., 2022; Melo et al., 2023)?

In this bibliometric analysis, we provide a comprehensive overview of the role of PTEN in the nervous system, particularly in neural development and neurological diseases. First, we determined the distribution and frequency of related publications and citations. Additionally, we reviewed the most influential research results and achievements associated with PTEN in the nervous system. These results can help new researchers develop their research interests and gain basic information. Moreover, our findings may provide precise clinical guidelines and strategies for treating nervous system injuries and diseases caused by PTEN dysfunction.

## 2 Data and methods

### 2.1 Data strategy and screening criteria

We first retrieved similar keywords in MeSH Database^1^ and the Web of Science Core Collection (WoSCC) following the retrieved formula: #1: (((TS = (phosphatase and tensin homolog deleted on chromosome ten)) OR TS = (PTEN)) OR TS = (MMAC1 Protein)) OR TS = (Mutated In Multiple Advanced Cancers 1 Protein); #2:(TS = (PTEN-induced kinase 1)) OR TS = (PINK1); #3:(TS = (Neuron)) OR TS = (Nerve); #4:(#1 NOT #2) AND #3.

**Figure 1:**
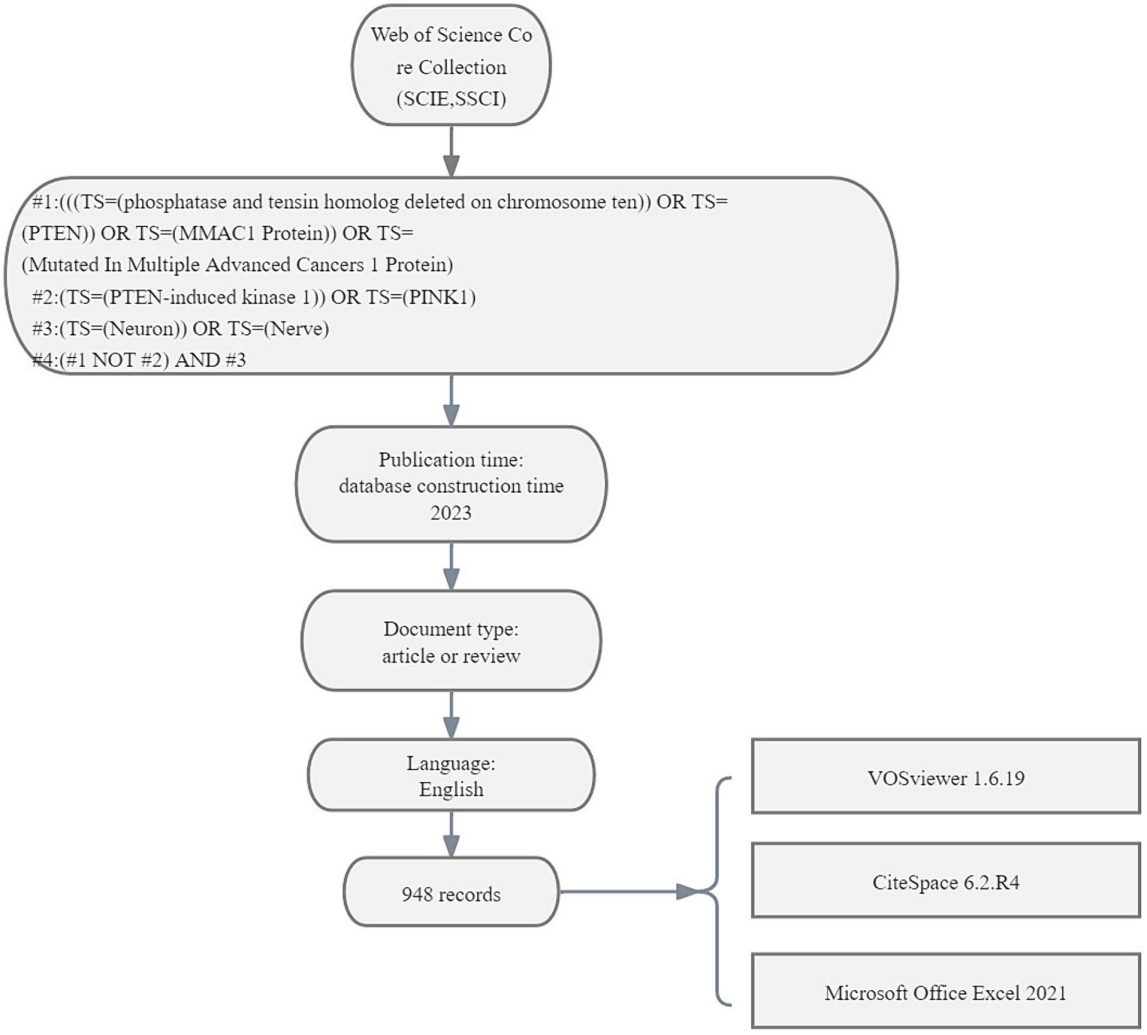
The search strategy. Flowchart of document selection.

We retrieved the literature from 1999 to 2023 and selected those listed as ‘Article’ and ‘Review,’ excluding letters, materials, books, corrections, and retracted literature. Next, two authors independently screened the literature based on the titles and abstracts to exclude studies unrelated to the topic of interest. This exclusion criterion was mainly used to limit the range of the authors, cited references, and keywords (Xu G et al., 2021; de Oliveira et al., 2023). If necessary, the authors read the full text to further screen for appropriate literature and discuss different views (Xu G et al., 2021; Wang et al., 2023). Finally, we obtained and analysed 948 documents. All the records of the retrieved files were downloaded and saved in text form (Figure 1). This retrieval was completed on 23 January 2024.

### 2.2 Methodology

Bibliometric analysis is a data analysis method that uses CiteSpace and VOSviewer, which applies artificial intelligence, mathematics, and statistics to study the change patterns, quantitative relationships, and distribution structure of the literature (Wang and Maniruzzaman, 2022; Guo J et al., 2023). The bibliometric research results include, but are not limited to, the publication year, authors, institutions, countries, journals, cited references, and keywords (Wang and Maniruzzaman, 2022; van den Hoven et al., 2023). All retrieved and downloaded information was collated into VOSviewer and CiteSpace for scientific mapping and visualization (Zhang T et al., 2023).

**Figure 2:**
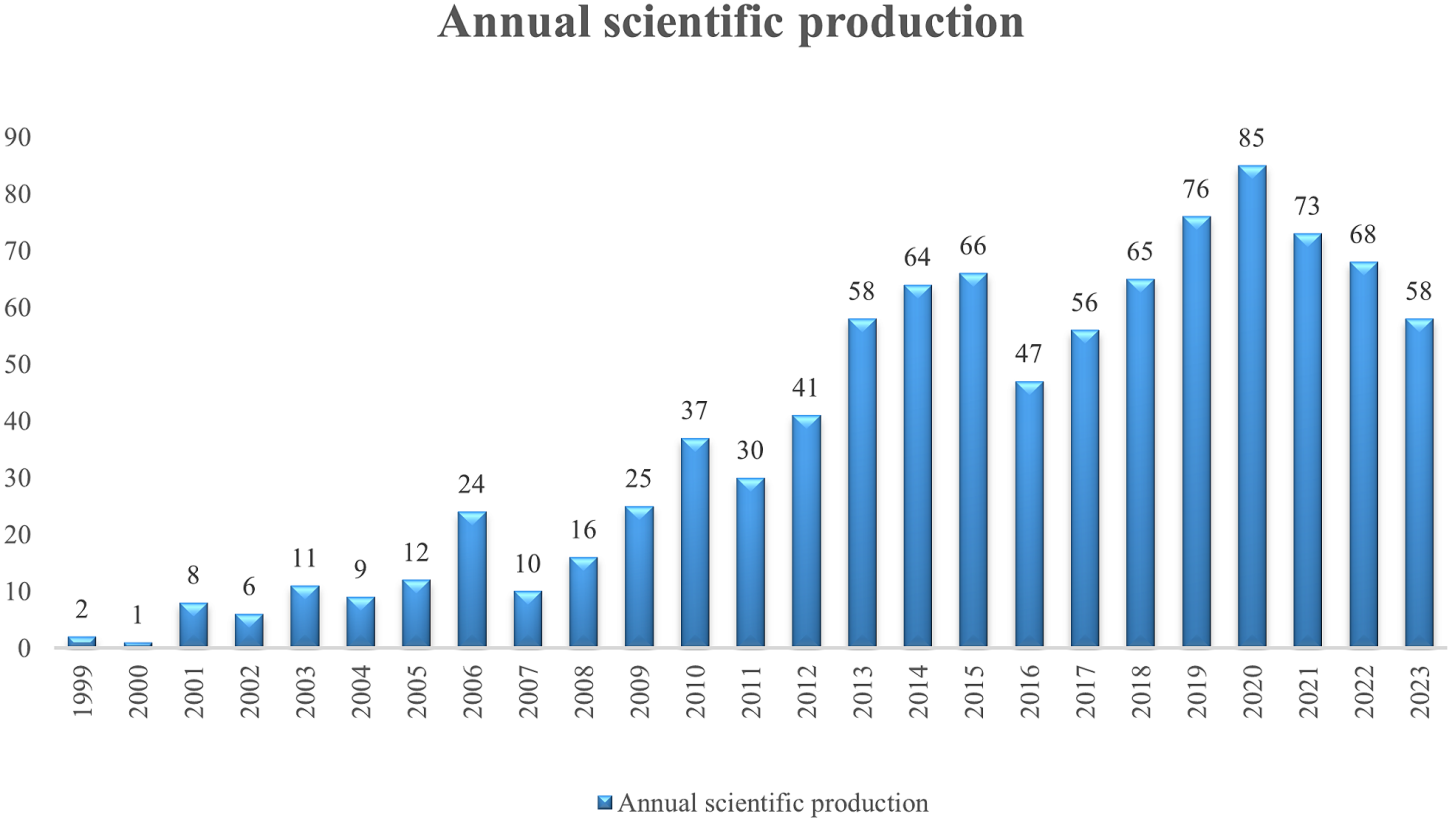
Publication outputs over the years on PTEN in the nervous system. The literature numbers showed an increasing but unstable trend.

Using CiteSpace, citations can be visualized by focusing on potential scientific areas (Zhu et al., 2021; Albuquerque et al., 2022). The software is based on scientific metrics and information visualization. It can draw and visualize a co-occurrence map of cooperation among authors, institutions, and countries with top citations and keyword clustering (Peng et al., 2022; Tan et al., 2023). Using VOSviewer, we constructed bibliometric networks based on authors, journals, and individual publications (Tan et al., 2023). It was also used to visualize and analyse the co-occurrence networks of key research terms, ultimately allowing us to gain a comprehensive understanding of the development of the research area (Albuquerque et al., 2022; Peng et al., 2022). Finally, the number of publications and published information (Figure 2) were analysed and drew using Microsoft Office Excel.

## 3 Results

### 3.1 Publication outputs

To evaluate the research progress in the field of PTEN in the nervous system, we retrieved the publication outputs of 881 studies from the WoSCC over the years (Figure 2). Overall, the literature shows an increasing but unstable trend. From 1999 (*n* = 2), the number of studies increased and peaked in 2006 (*n* = 24), but declined in 2007 (*n* = 10). From 2007, the number of studies increased again and peaked in 2015 (*n* = 66) but declined in 2016 (*n* = 47). Finally, from 2016, the number of studies increased and peaked in 2020 (*n* = 85) but decreased annually from 2020. Nonetheless, the overall increasing trend suggests that research on PTEN expression in the nervous system has drawn increasing attention in recent years.

### 3.2 Distribution of studies by country or region

**Figure 3:**
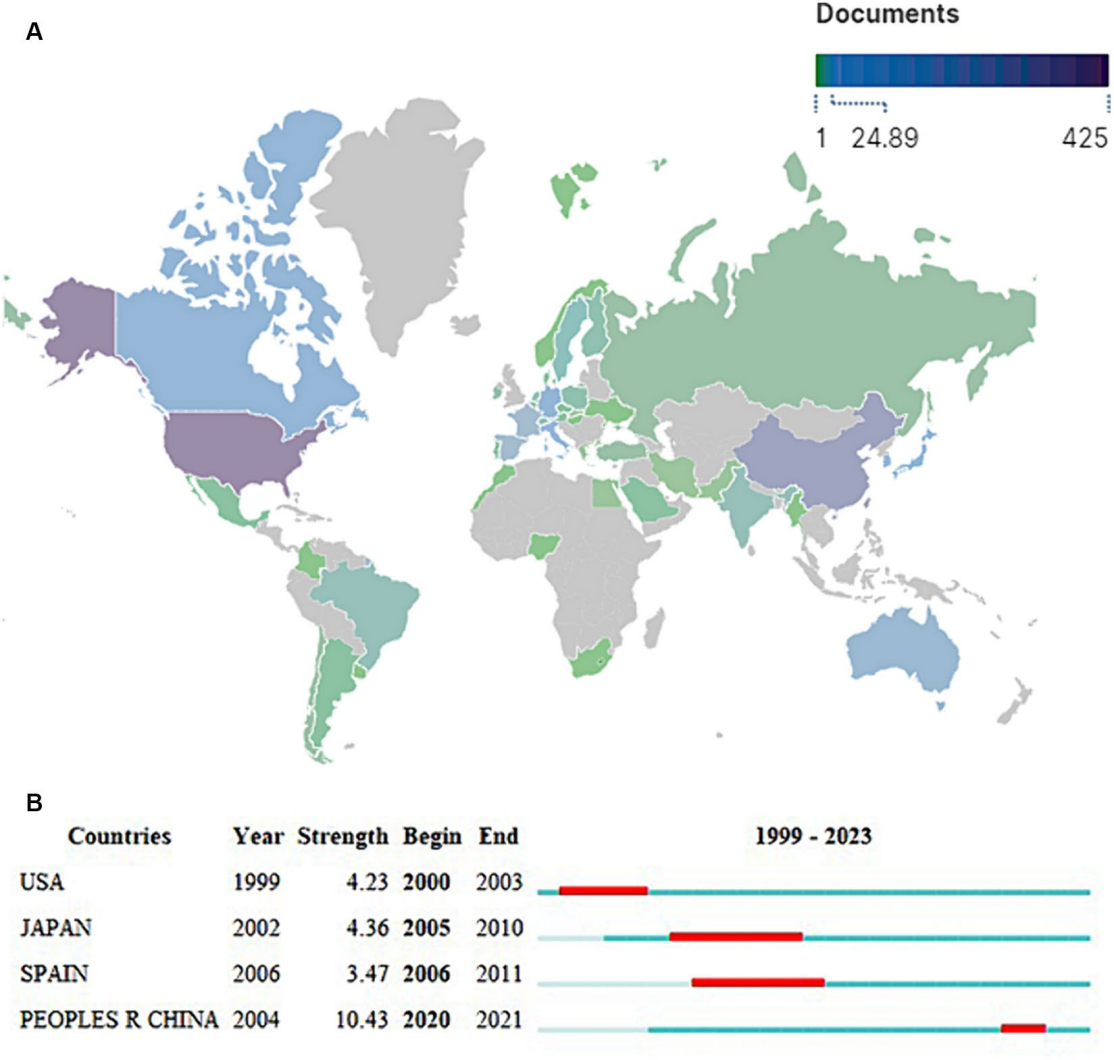
Distribution of studies by countries or regions. **(A)** Word map of the spatial distribution of the literature. The number of studies is indicated by distinct colors, while grey represents no publications from that country. The United States published the most studies with the highest number of citations. **(B)** The top four countries with the strongest citation bursts. The red bars represent citation burst durations.

Forty-seven countries/regions had contributed to the study of PTEN in the nervous system (Figure 3A). The top 10 countries/regions that published the most studies are shown in Table 1, including the United States (*n* = 425), China (*n* = 286), England (*n* = 59), Canada (*n* = 58), and Germany (*n* = 57). Although China has ranked among the top two productive countries, the total and average citations are lower compared with other countries/regions with fewer studies (Figure 3A; Table 1). A strong burst of citations can be reflected in changes over a short period. The country with the highest burst strength was China (10.43), followed by Japan (4.36) (Figure 3B), indicating that many researchers from China and Japan are devoted to studying PTEN in the nervous system.

**Table 1:** Top 10 most productive countries/regions.

Rank	Country/Region	Publications	Citations	Average citation/Publication
1	United States	425	27,823	65.47
2	China	286	7,814	27.32
3	England	59	3,001	50.86
4	Canada	58	2,795	48.19
5	Germany	57	2,611	45.81
6	Japan	41	1,947	47.49
7	Italy	25	1,003	40.12
8	South Korea	25	657	26.28
9	Australia	20	615	30.75
10	Spain	19	647	34.05

### 3.3 Co-authorship of institutions

**Figure 4:**
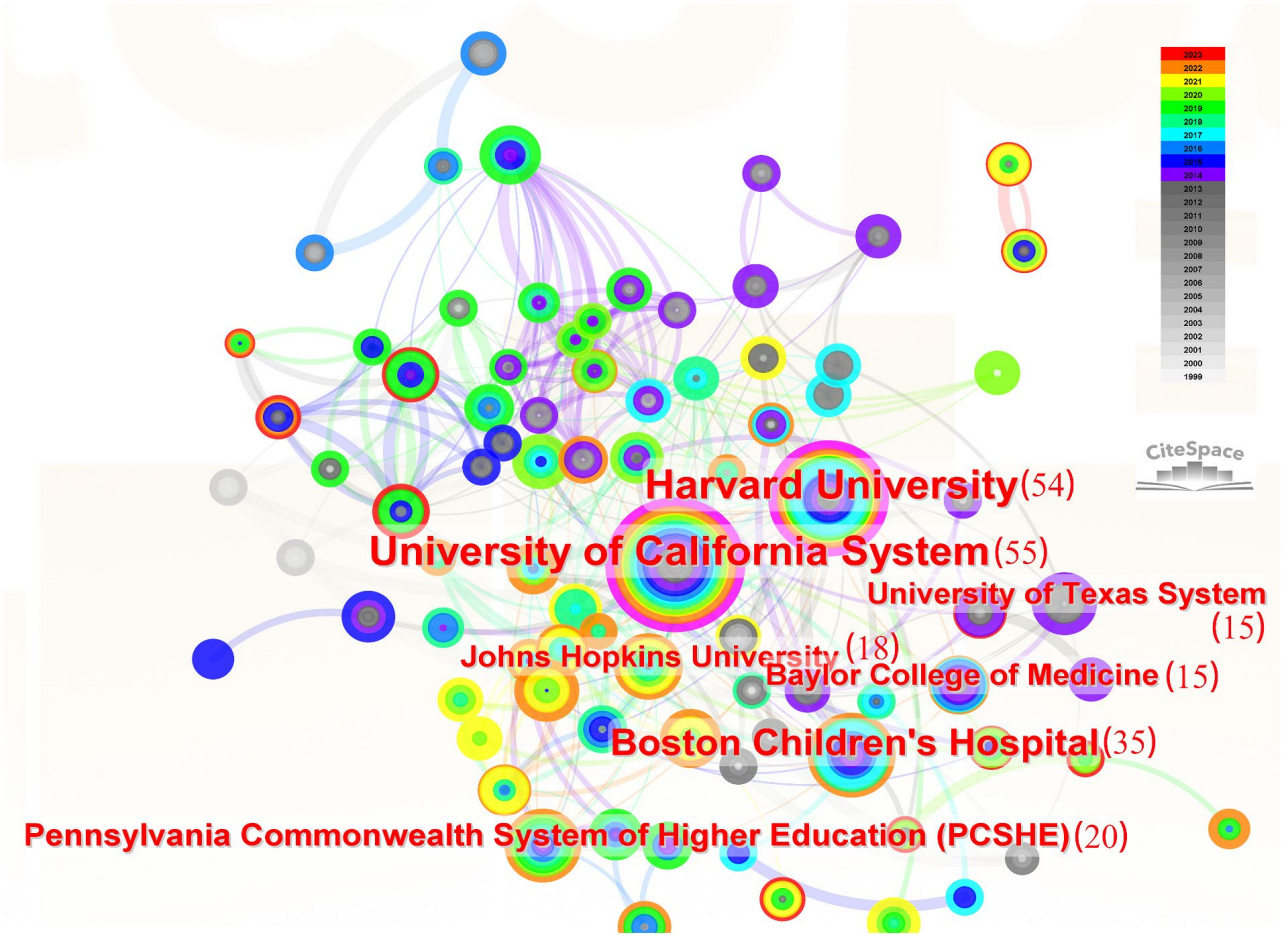
Co-authorship of institutions. The most prolific institutions, which collaborate more with others, are from the United States. The numbers of studies are in brackets. The width of the line indicates the strength of the relationship.

A total of 1,037 institutions contributed to research on PTEN in the nervous system. The top seven institutions that published the most literature are labelled in Figure 4, including the University of California System (55 documents), Harvard University (54 documents), Boston Children’s Hospital (35 documents), Pennsylvania State System of Higher Education (20 documents), Johns Hopkins University (18 documents), the University of Texas System (15 documents), and Balor College of Medicine (15 documents), indicating that the most influential institutions in the field are from the United States. We also visualized the institutions with more than five studies (Figure 4). The partnership network results showed that all the most prolific institutions were from the United States, indicating that those institutions may make up the core of research in this field. Of note, the only non-American institutions among the top 10 were Shanghai Jiao Tong University, Nantong University of China, and the University of London of England, indicating that institutions from other countries/regions should strengthen their cooperation with other institutions (Figure 4).

### 3.4 Co-authorship of authors

**Figure 5:**
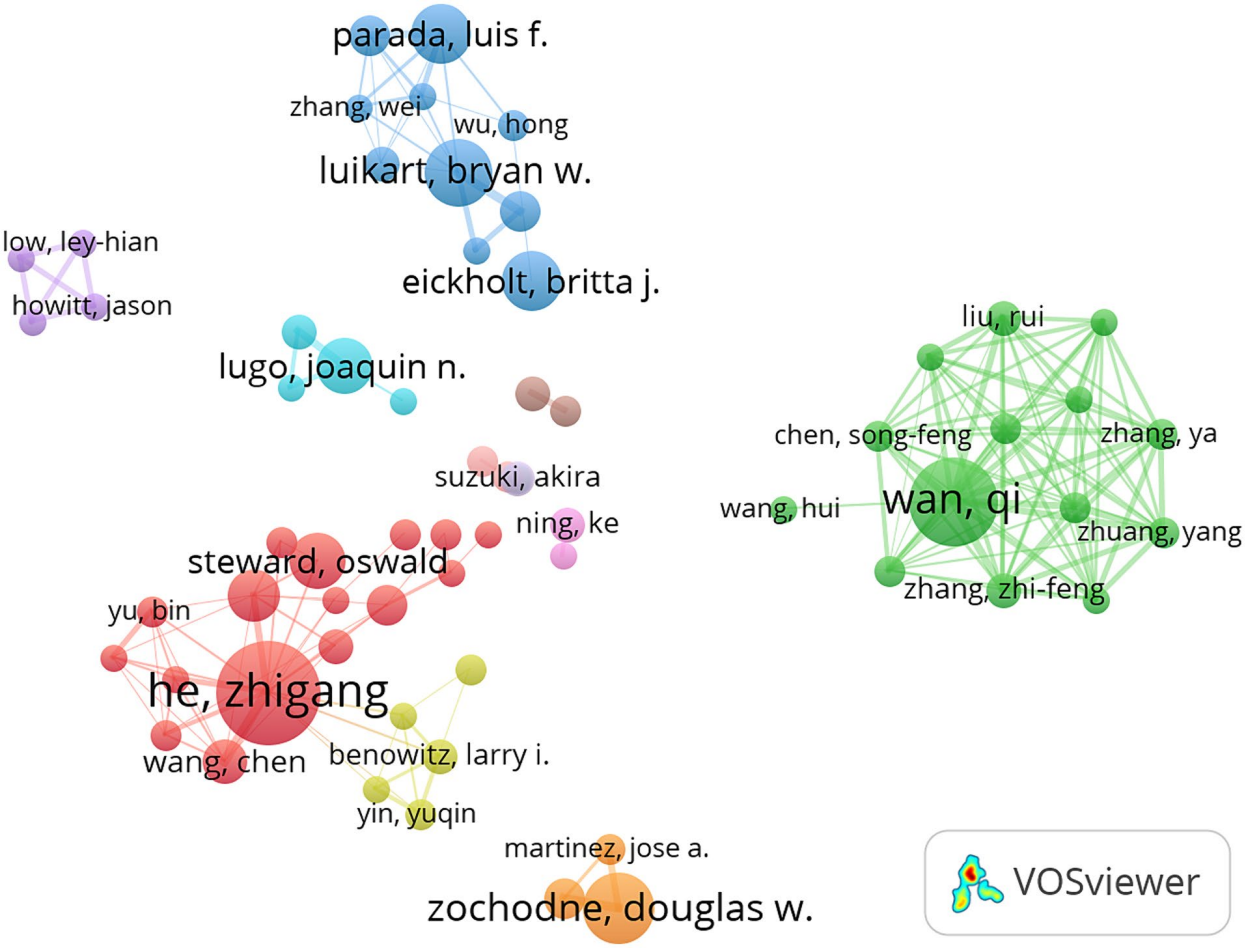
Co-authorship of authors. Half of the top 10 authors are from the United States. The lines between the nodes indicate collaborations among the authors. The line widths indicate the strength of the relationship.

A total of 5,398 authors participated in research on PTEN in the nervous system. The top 10 authors who published the greatest quantity of studies are listed in Table 2. Half of the top 10 authors were from the United States, indicating that these authors might have the greatest influence in the related field. Zhigang He (22 documents), from Harvard University, is the most prolific author, who is mainly devoted to investigating effective and translatable axon regeneration methods for nervous injuries (Cartoni et al., 2017; Norsworthy et al., 2017; Jacobi et al., 2022), indicating that research on PTEN in axon regeneration is a hot research topic. Qi Wan (21 documents) is a specialist leading in the research of PTEN-mediated neuronal death from Qingdao University and worked at the Toronto Western Research Institute (Ning et al., 2004; Liu B et al., 2010; Kong et al., 2022). Prof. Wan revealed a series of comprehensive neuroprotective mechanisms mediated by PTEN-related pathways in brain injuries and neurodegenerative diseases, making PTEN-mediated neuronal death another popular research topic (Chang et al., 2007; Hu et al., 2023; Kong et al., 2023). Next, we set the threshold to four studies and analysed the authors who met the criteria (Figure 5). In the visualized map, the lines between the nodes indicate collaboration among authors. Overall, there are 11 color clusters, of which the top three are centred on Zhigang He, Qi Wan, and Douglas W. Zochodne, indicating that they are the most influential authors in this research area. However, there were almost no connections among the different clusters, indicating that the authors collaborated very little in the different sub-research areas.

**Table 2:** Top 10 authors with most publications.

Rank	Authors	Institutions (Countries/Regions)	Publications	Citations
1	He, Zhigang	Harvard University (United States)	25	4,330
2	Wan, Qi	Qingdao University (China)	21	434
3	Zochodne, Douglas W	University of Alberta (Canada)	16	547
4	Luikart, Bryan W	Dartmouth College (United States)	15	1,121
5	Parada, Luis F	Memorial Sloan Kettering Cancer Center (United States)	13	2097
6	Eickholt, Britta J	Charité Universitätsmedizin Berlin (Germany)	13	461
7	Lugo, Joaquin N	Baylor University (United States)	12	436
8	Steward, Oswald	University of California at Irvine (United States)	12	956
9	Liu, Kai	The Hong Kong University of Science and Technology (China)	11	2,623
10	Wang, Chen	Peking University (China)	9	1913

### 3.5 Density visualization of journals

Literature on PTEN in the nervous system has been published in 337 journals. By analysing journals that published more than five articles, we found that the *Journal of Neuroscience* was the top journal publishing on this topic and had the highest total and average citations, making it the most influential journal in this research field (Table 3). Our results also indicate that the top 10 journals with the highest impact factor in 2021 ranged from 2.5 to 11.1, and *Proceedings of the National Academy of Sciences of the United States of America (PNAS)* had the highest impact factor.

**Table 3:** Top 10 journals with most publications.

Rank	Source	Publications	Citations	Average Citation/Publication	2022 Impact Factor	2022 JCR Partition
1	Journal of Neuroscience	54	4,998	92.56	5.3	Q1
2	Experimental Neurology	31	1,007	32.48	5.3	Q2
3	PLoS One	26	742	28.54	3.7	Q2
4	Proceedings of the National Academy of Sciences of the United States of America	19	1,479	77.84	11.1	Q1
5	Neurobiology of Disease	17	405	23.82	6.1	Q1
6	Neuroscience	15	793	52.87	3.3	Q3
7	Neural Regeneration Research	15	167	11.13	6.1	Q1
8	Cell Reports	15	584	38.93	8.8	Q1
9	Neuroscience Letters	14	315	22.5	2.5	Q3
10	Scientific Reports	14	210	15	4.6	Q2

### 3.6 Co-authorship of top-cited literature and co-citation analysis

**Figure 6:**
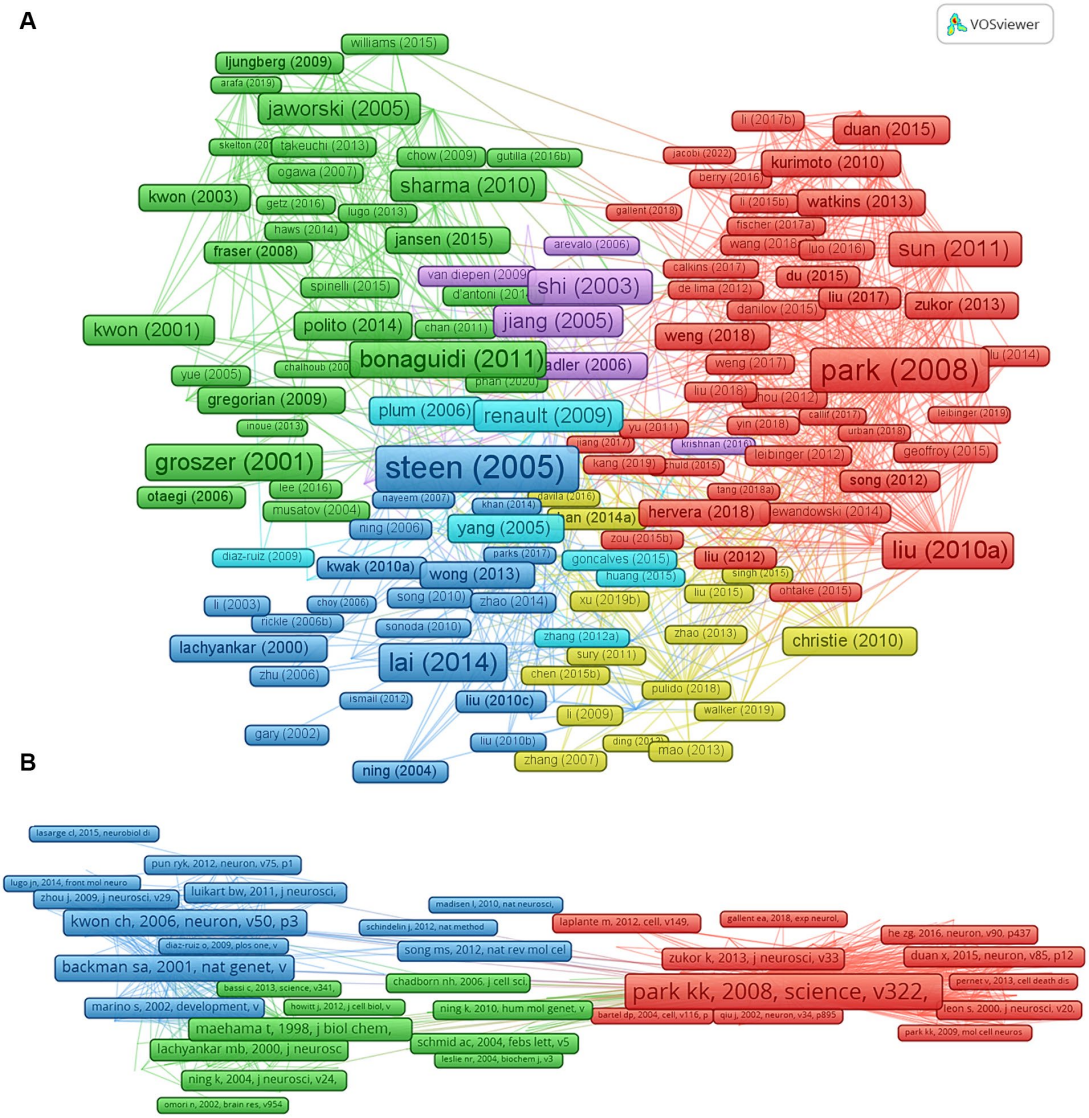
Co-authorship of top-cited literature and co-citation analysis. **(A)** Studies that have been cited more than 10 times. **(B)** The top 10 co-cited references. The same color clusters indicate the documents are related or have some commonalities. The lines between the nodes indicate the connections among the documents. The top three co-cited references are also involved in the top 10 cited studies.

Literature cited more than 10 times was analysed and is listed (Figure 6A; Table 4). These findings may indicate the most influential studies associated with PTEN in the nervous system. Among them, ‘Impaired insulin and insulin-like growth factor expression and signaling mechanisms in Alzheimer’s disease - is this type 3 diabetes?’ (Steen et al., 2005), published in *Journal of Alzheimer’s Disease* in 2005, has been most cited (1,266 Citations), followed by ‘Promoting Axon Regeneration in the Adult CNS by Modulation of the PTEN/mTOR Pathway’ (Park et al., 2008), published in *Science* in 2008 (1,139 Citations), and ‘PTEN deletion enhances the regenerative ability of adult corticospinal neurons’ (Liu K et al., 2010), published in *Nature Neuroscience* in 2010 (668 Citations). Besides, ‘Excitotoxicity and stroke: Identifying novel targets for neuroprotection’ (Lai et al., 2014), published in *Progress in Neurobiology* in 2014, is the most cited review (709 Citations).

**Table 4:** Top 10 most cited publications.

Rank	Title	First author	Type	Source	Year	Citations
1	Impaired insulin and insulin-like growth factor expression and signaling mechanisms in Alzheimer’s disease - is this type 3 diabetes?	E Steen	Article	Journal of Alzheimer’s Disease	2005	1,266
2	Promoting Axon Regeneration in the Adult CNS by Modulation of the PTEN/mTOR Pathway	Kevin Kyungsuk Park	Article	Science	2008	1,139
3	Excitotoxicity and stroke: Identifying novel targets for neuroprotection	Ted Weita Lai	Review	Progress in Neurobiology	2014	709
4	PTEN deletion enhances the regenerative ability of adult corticospinal neurons	Kai Liu	Article	Nature Neuroscience	2010	668
5	Negative regulation of neural stem/progenitor cell proliferation by the PTEN tumor suppressor gene *in vivo*	M Groszer	Article	Science	2001	638
6	*In vivo* Clonal Analysis Reveals Self-Renewing and Multipotent Adult Neural Stem Cell Characteristics	Michael A Bonaguidi	Article	Cell	2011	590
7	Sustained axon regeneration induced by co-deletion of PTEN and SOCS3	Fang Sun	Article	Nature	2011	505
8	Hippocampal neuronal polarity specified by spatially localised mPar3/mPar6 and PI 3-kinase activity	SH Shi	Article	Cell	2003	493
9	Control of dendritic arborization by the phosphoinositide-3′-kinase-Akt-mammalian target of rapamycin pathway	J Jaworski	Article	Journal of Neuroscience	2005	451
10	Both the establishment and the maintenance of neuronal polarity require active mechanisms: critical roles of GSK-3 beta and its upstream regulators	H Jiang	Article	Cell	2005	443

We further visualized the co-cited references and listed the top ten articles using VOSviewer (Figure 6B; Table 5), which can indicate the core references cited by other studies in the same research area. ‘Promoting Axon Regeneration in the Adult CNS by Modulation of the PTEN/mTOR Pathway’ has the most citations (224 Citations) (Park et al., 2008), followed by ‘PTEN deletion enhances the regenerative ability of adult corticospinal neurons’ and ‘Sustained axon regeneration induced by co-deletion of PTEN and SOCS3’ (Liu K et al., 2010). Of note, the top three co-cited references were also included in the top ten cited studies, indicating that they are also the most influential studies in the research field.

**Table 5:** Top 10 co-cited references.

Rank	Title	First author	Type	Source	Year	Citations
1	Promoting Axon Regeneration in the Adult CNS by Modulation of the PTEN/mTOR Pathway	Kevin Kyungsuk Park	Article	Science	2008	224
2	PTEN deletion enhances the regenerative ability of adult corticospinal neurons	Kai Liu	Article	Nature Neuroscience	2010	158
3	Sustained axon regeneration induced by co-deletion of PTEN and SOCS3	Fang Sun	Article	Nature	2011	110
4	PTEN regulates neuronal arborization and social interaction in mice	Chang-Hyuk Kwon	Article	Neuron	2006	109
5	Deletion of PTEN in mouse brain causes seizures, ataxia and defects in soma size resembling Lhermitte-Duclos disease	S. A. Backman	Article	Nature genetics	2001	102
6	PTEN regulates neuronal soma size: a mouse model of Lhermitte-Duclos disease	C. H. Kwon	Article	Nature genetics	2001	99
7	PTEN Inhibition to Facilitate Intrinsic Regenerative Outgrowth of Adult Peripheral Axons	Kimberly J. Christie	Article	Journal of Neuroscience	2010	97
8	The tumor suppressor, PTEN/MMAC1, dephosphorylates the lipid second messenger, phosphatidylinositol 3,4,5-trisphosphate	T. Maehama	Article	Journal of Biological Chemistry	1998	92
9	Negative regulation of neural stem/progenitor cell proliferation by the PTEN tumor suppressor gene *in vivo*	M. Groszer	Article	Science	2001	82
10	PTEN/mTOR and axon regeneration	Kevin K. Park	Review	Experimental Neurology	2010	69

### 3.7 The analysis of the current research hotpots

**Figure 7:**
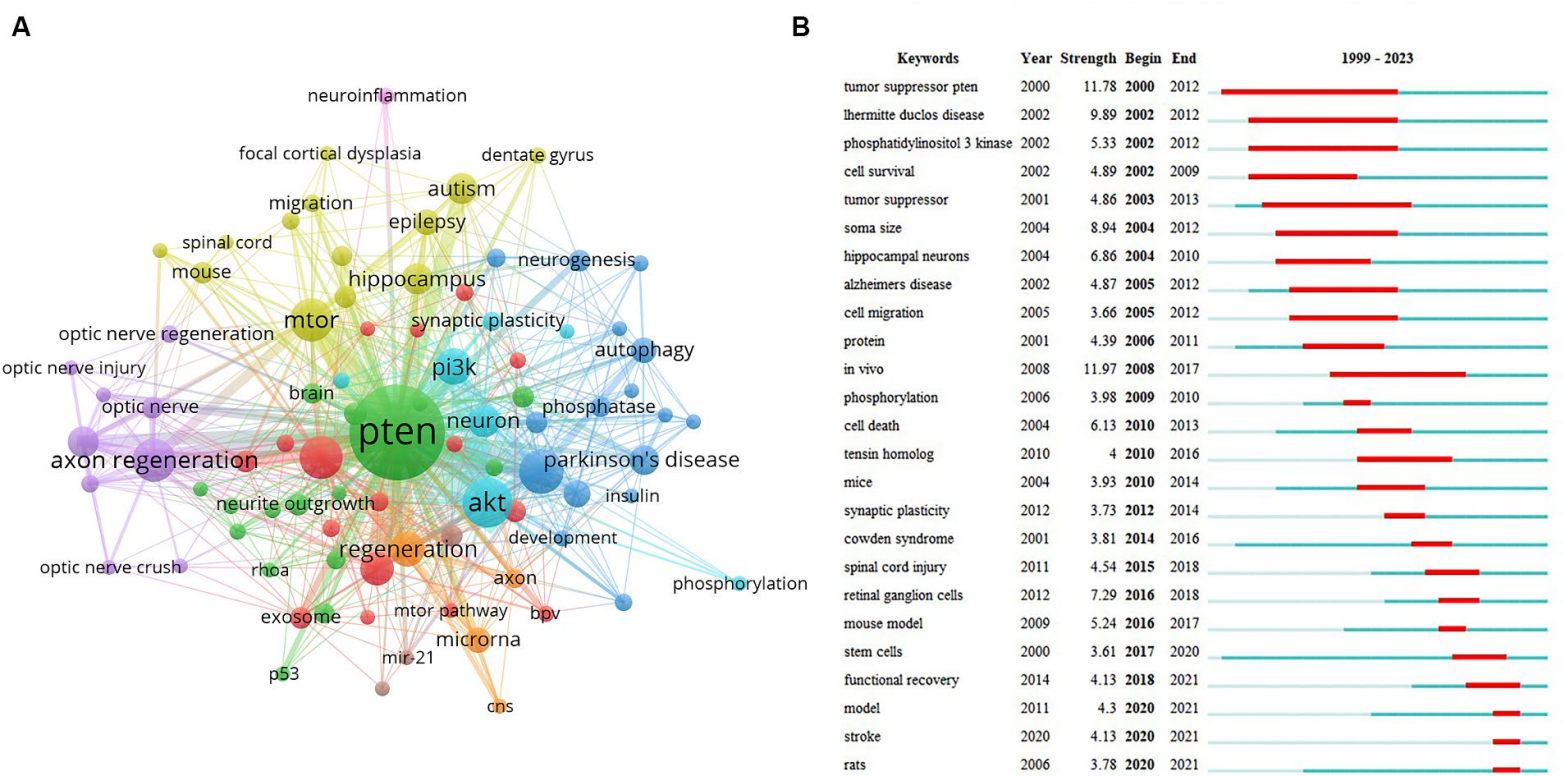
Analysis of the current research hotspots. **(A)** Top cited keywords with more than five studies. The circle size indicates the frequency. The width of the line indicates the strength of the relationship. **(B)** Top 25 keywords with the strongest citation bursts. The red bars represent citation burst durations.

The top-cited keywords over the years indicate the current research trends in the research area. In this section, we visualized keywords with more than five articles (Figure 7A). Keywords such as PTEN (256 times), Akt (64 times), mTOR (46 times), axon regeneration (46 times), and spinal cord injury (46 times) appeared to be cited most frequently, indicating current research hotspots (Figure 7A).

Citation explosion, indicated by cited burst strength, refers to the rapid increase in the number of cited keywords in a brief period. This indicates that keywords have drawn significant attention in this field during this period. The top 25 keywords with the highest cited burst strengths were further analysed (Figure 7B). ‘*In vivo*’ had the strongest burst strength of 11.97 (Figure 7B), suggesting that ‘*in vivo*’ study about PTEN in the nervous system is the key hotspot. In addition, nervous system diseases include Lhermitte–Duclos disease, Alzheimer’s disease (AD), spinal cord injury (SCI), and stroke. Cellular mechanisms, such as cell death, migration, and synaptic plasticity, which have strong burst strength, also occurred in the top 25 keywords (Figures 7A,B), suggesting that they are also key research hotspots.

Finally, we specifically analysed the primary molecules involved in the research on PTEN in the nervous system, which could suggest the key regulated pathways involved in the related research area. The top 15 molecules with the highest frequencies are listed in Table 6, mainly covering PTEN (256 times), Akt (64 times), mTOR (46 times), PI3K (32 times), and NMDA receptor (6 times). We also listed the most frequently occurring cellular mechanisms, including apoptosis (48 times), axon regeneration (46 times), autophagy (15 times), inflammation (12 times), and differentiation, neurogenesis, and oxidative stress (11 times each). These results suggest that cellular mechanisms have attracted considerable attention as a potential research hotspot. Table 6 lists the pathological diseases involving the study of PTEN in the nervous system. The 15 most frequently occurring diseases were SCI (46 times), autism (22 times), Parkinson’s disease (PD) (20 times), AD (17 times), and epilepsy (15 times), which are the most common diseases studied in relation to PTEN involvement in the nervous system.

**Table 6:** Top 15 signaling molecules, cellular mechanisms and pathological diseases.

Rank	Signaling molecules	Occurrences	Cellular mechanisms	Occurrences	Pathological diseases	Occurrences
1	PTEN	256	Apoptosis	48	Spinal cord injury	46
2	akt	64	Axon regeneration	46	Autism	22
3	mTOR	46	Autophagy	15	Parkinson’s disease	20
4	PI3K	32	Inflammation	12	Alzheimer’s disease	17
5	NMDA receptor	6	Differentiation	11	Epilepsy	15
6	micrna-21	6	Neurogenesis	11	Aging	10
7	Dj-1	5	Oxidative stress	11	Stroke	10
8	SOCS3	5	Proliferation	9	Diabetes	8
9	mir-21	5	Synaptic plasticity	8	Amyotrophic lateral sclerosis	7
10	tau	5	Migration	7	Ischemia	6
11	RhoA	5	Development	6	Glioblastoma	6
12	P53	5	Myelin	6	Optic nerve injury	5
13	EGFR	5	Neurotoxicity	6	Peripheral nerve injury	5
14	insulin	5	Phosphorylation	5	Focal cortical dysplasia5	5
15	PDK1	4	Cell polarity	5	Traumatic brain injury	5

### 3.8 The analysis of the future research trends

**Figure 8:**
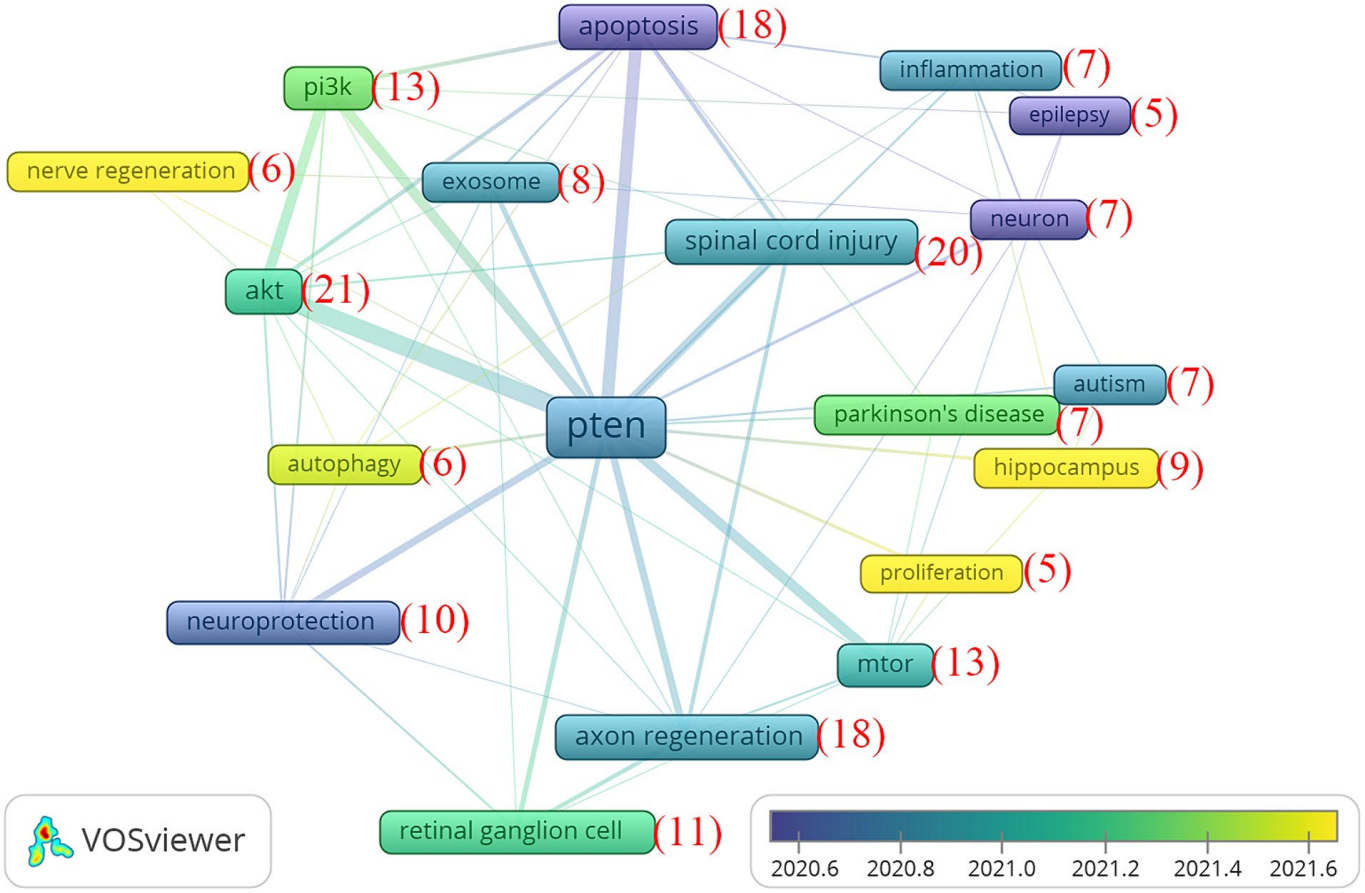
Analysis of the future research trends. Top cited keywords with more than five studies published in 5 years. The numbers of citations are in brackets. The width of the line indicates the strength of the relationship.

Analysing the publication trends of PTEN in the nervous system published within the last 5 years could allow further extrapolation of the future trends of PTEN research. Therefore, we visualized and listed the top emerging research keywords with more than five articles, including those regarding signaling molecules, cellular mechanisms, and diseases (Figure 8). The frequency figures are labelled in Figure 8, mainly covering the signaling molecules, such as akt (21 times), PI3K (13 times), and mTOR (13 times), the cellular mechanisms, such as apoptosis (18 times), axon regeneration (18 times) and inflammation (7 times), and diseases including spinal cord injury (20 times), Parkinson’s disease (7 times) and autism (7 times), which may be hotspots and targets of further research trends on the study of PTEN in the nervous system.

### 3.9 An overview of signaling molecules, cellular mechanisms, and pathological diseases

**Figure 9:**
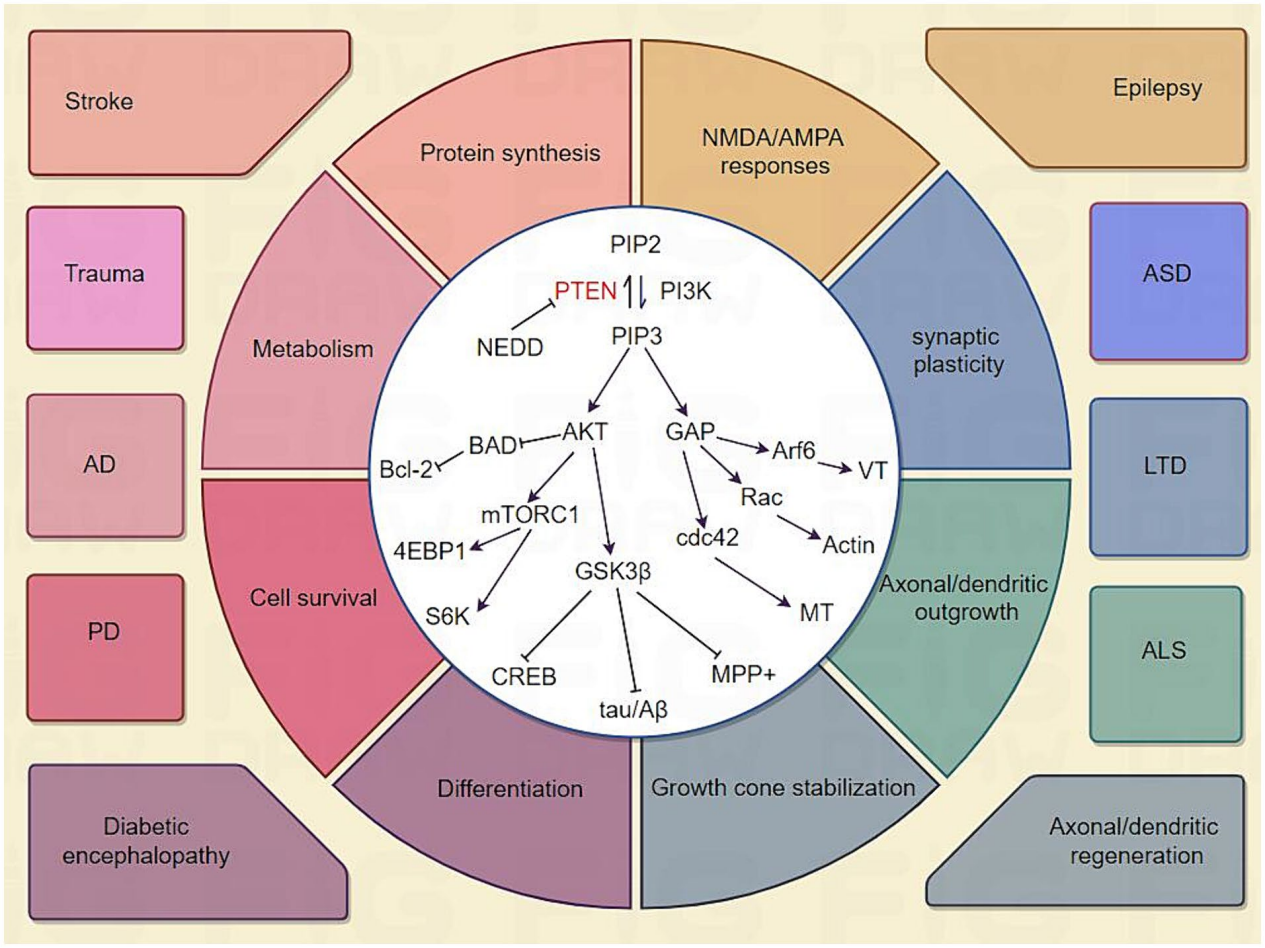
Diagram of the common PTEN and downstream pathways in the nervous system. Canonical PTEN signaling cascade. Activation is depicted with pointed arrows, and inhibition is depicted with flat-headed arrows. PTEN, phosphatase and tensin homolog deleted on chromosome ten; PI3K, phosphoinositide 3-kinase; PIP2, phosphatidylinositol 4,5-bisphosphate; PIP3, phosphatidylinositol 3,4,5-trisphosphate; AKT, protein kinase B; BAD, Bcl2-Associated Agonist Of Cell Death; mTORC1, mammalian target of rapamycin complex 1; Bcl-2, B-cell lymphoma-2; 4EBP1, eIF4E-binding protein; S6K, ribosomal S6 kinase; GSK3, glycogen synthase kinase3; CREB, cAMP-response element binding protein; Aβ, amyloid β-protein; MPP+, 1-methyl-4-phenylpyridinium; GAPs, GTPase activating proteins; cdc42, cell division cycle 42; Arf6, ADP-ribosylation factor 6; MT, microtubule; VT, vesicle trafficking; NEDD4, neural precursor cell expressed developmentally down-regulated protein 4; AD, Alzheimer’s disease; SCI, spinal cord injury and stroke; PD, Parkinson’s disease; ASD, autism spectrum disorders; LTD, long-term depression.

In this section, we review the key signaling molecules, cellular mechanisms, and pathological diseases involved in research on PTEN and the nervous system (Figure 9). Many studies have indicated that PTEN is the main antagonist of PI3K/Akt signaling by dephosphorylating PIP3 to PIP2 (Li et al., 2021; Nguyen Huu et al., 2021). PIP3 activation after PTEN inhibition could then activate Akt, which is the most prominent PIP3 effector (Li et al., 2021; Dibble et al., 2022). Akt is thought to be the main regulatory molecule involved in cell survival, growth, and proliferation through the phosphorylation and regulation of a series of protein activities (Hoxhaj and Manning, 2020). For example, Akt can phosphorylate and activate the mTOR complex, which promotes protein synthesis and cell proliferation (Li et al., 2013). Akt could also phosphorylate but inactivate glycogen synthase kinase3 (GSK3) (Jiang et al., 2005). This is a vital part of a series of neuronal responses and impairment that occurs by regulating cAMP-response element binding protein (CREB) (Zhou et al., 2014; Zeng et al., 2022), amyloid β-protein (Aβ) (Li et al., 2016; Wani et al., 2019), 1-methyl-4-phenylpyridinium (MPP+) (Guo S et al., 2023; Yu et al., 2023), etc. Besides Akt, PIP3 recruits several GTPase-activating proteins (GAPs) (Shen et al., 2017; Zhu et al., 2019) to the membrane and further activates GTPase families, such as Rac, cdc42, and Arf (Sakakibara and Horwitz, 2006; Zheng et al., 2008; Kilic et al., 2010; Kreis et al., 2014), ultimately modulating cytoskeletal movement and vesicular transport at the membrane, which are important in synaptic potentiation, neurite outgrowth, axon and dendrite specifications, and regeneration (Zheng et al., 2008; Rademacher and Eickholt, 2019; Nieuwenhuis and Eva, 2022). In addition to regulating protein synthesis, the PTEN/PI3K/Akt pathway is important for neuronal survival by regulating BAD/Bcl-2 (Hua et al., 2016). Upstream of PTEN, a neural precursor cell expressing developmentally downregulated protein 4 (NEDD4) functions as an impeder of axon regeneration by catalyzing PTEN ubiquitination and degradation (Dai et al., 2021; Sun et al., 2022).

## 4 Discussion

In this study, we analysed the current hotspots and future trends in the study of PTEN in the nervous system, especially in neural development and pathological diseases, using bibliometrics. We first found that the number of publications tended to grow with time, but it was not stable. Universities, institutions, and authors from the United States are leading researchers in this area. Finally, many cutting-edge research results have been discovered, such as key regulatory molecules and cellular mechanisms of PTEN in the nervous system, which could provide novel intervention targets and precise therapeutic strategies for related injuries and diseases.

### 4.1 General information

This study retrieved 948 papers published between 1999 and 2023 from WoSCC. Overall, many studies have demonstrated the development and importance of PTEN in the nervous system. Presently, the United States, China, England, Canada, and Germany are in the top five, with the highest number of publications or citations and the strongest influence. Institutions from the United States, such as the University of California System, Harvard University, Boston Children’s Hospital, Pennsylvania State System of Higher Education, and Johns Hopkins University, have published the most documents, indicating that the United States is leading the research and might be the core of research in the world. Additionally, China had the strongest citation bursts in the world, indicating that more researchers in China are devoted to researching the role of PTEN in the nervous system. Regarding journal quality, *PNAS* had the highest impact factor among journals that have published more than five studies. The *Journal of Neuroscience* published the most literature and is the most influential journal in this research area.

### 4.2 High-impact literature

In 2005, Dr. Eric Steen published one article, ‘Impaired insulin and insulin-like growth factor expression and signaling mechanisms in Alzheimer’s disease - is this type 3 diabetes?’ in the *Journal of Alzheimer’s Disease*, which had the most citations. This study found that deficient energy metabolism and glucose utilization occurred during early AD. They further discussed the impaired insulin signaling in AD, which is accompanied by reduced levels of tau, PI3K, Akt, enhanced glycogen synthase kinase-3β, PTEN, and Aβ production (Steen et al., 2005). Dr. Zhigang He has published 25 papers, making him the leading author in the research on PTEN in the nervous system. The most cited research article from Dr. Zhigang He is ‘Promoting Axon Regeneration in the Adult CNS by Modulation of the PTEN/mTOR Pathway,’ which was published in *Science*. In this study, they found that there are intrinsic inhibitory molecules impeding axon regeneration and that mTOR activation and protein production were inhibited in crushed retinal ganglion cell (RGC) axons, which may further inhibit axon regrowth. However, the deletion of PTEN in adult RGCs using a conditional knockout approach can robustly facilitate axon regeneration. Thus, their findings provided a manipulated therapeutic method for the intrinsic pathway to enhance axon regrowth after the crushing of axons (Park et al., 2008).

The co-citation map indicates intensive cooperation among researchers interested in the same research area. We found that another two of the top three co-cited references, ‘PTEN deletion enhances the regenerative ability of adult corticospinal neurons’ and ‘Sustained axon regeneration induced by co-deletion of PTEN and SOCS3’, are also involved in the top 10 cited studies. The first study indicates that the PTEN/mTOR pathway is critical for promoting corticospinal tract axon regeneration in injured corticospinal neurons (Liu K et al., 2010). The second study showed that knockout of both PTEN and suppressor of cytokine signaling 3 (SOCS3) cooperatively enhance robust axon regrowth by concurrent activation of mTOR and the Janus kinase (JAK)-signal transducers and activators of transcription (STAT) pathway (Sun et al., 2011). Thus, these highly cited and co-cited articles all proved that targeting intrinsic PTEN is an effective method for enhancing axon regrowth after axonal injury, indicating that it is also a hot topic in the research field.

### 4.3 Hotspots and frontiers

A keyword frequency analysis was used to reflect the current trends and future directions of a research field. In this study, we found that ‘*in vivo*’ has the highest burst strength, suggesting that *in vivo* studies of PTEN in the nervous system are the key hotspot. Some of the most frequent keywords were PTEN, Akt, mTOR, axon regeneration, and spinal cord injury, which are related to signaling molecules, cellular mechanisms, and diseases, suggesting that they are key research hotspots. Notably, the keyword frequency analysis results were consistent with the keywords used in highly cited literature.

To further clarify the specific hotspots in research on PTEN in the nervous system, we first analysed the key signaling molecules related to molecular biology and biochemistry. Except for the key regulators—such as PTEN, Akt, mTOR, and PI3K—exosomes, NMDA receptors, micRNA-21, and DJ-1 also appeared frequently, indicating key signaling molecules and hotspots of PTEN in the nervous system. Furthermore, analysis of the most frequently occurring cellular mechanisms, which mainly include apoptosis, axon regeneration, autophagy, inflammation, and differentiation/neurogenesis/oxidative stress, may provide hotspots for cellular mechanisms that have attracted considerable attention. Statistical analyses of key signaling molecules and cellular mechanisms indicated the importance of PTEN-mediated pathways, mechanisms, and phenotypes in the nervous system. Finally, we analysed the most frequently occurring diseases, mainly SCI, autism, PD, AD, epilepsy, ageing, and stroke, which are hotspot diseases in the study of PTEN involvement in the nervous system and revealed that these diseases attracted more attention among investigators.

Finally, we analysed the publication trends of PTEN in the nervous system published within the last 5 years, which could be used to extrapolate the future trends of PTEN research further. The most frequent keywords, mainly covering signaling molecules, such as Akt, PI3K, and mTOR; cellular mechanisms, such as apoptosis, axon regeneration, and inflammation; and pathological diseases, such as SCI, PD, and autism, suggest research trends in the study of PTEN involvement in the nervous system. However, we failed to analyse further and visualize the trends in potential therapeutics. In our analysis, we carefully screened potential therapeutics and found that no potential therapeutics appeared frequently, which means that no potentially effective or hotpot therapeutics are emerging, and the investigation of effective therapeutics for PTEN-mediated neurodegenerative diseases is urgently needed.

Taken together, a series of studies has suggested that PTEN is a key regulatory molecule in the nervous system, particularly in neural development and pathological diseases. By analysing important signaling molecules and cellular mechanisms, it is important to discover key regulated pathways and provide new therapeutic targets for PTEN-related nervous system injuries and diseases. In addition, discussing the key molecules, mechanisms, and diseases in the research of PTEN in the nervous system could provide wide insight into current research hotspots and could be a reference for new researchers to select their starting research directions. Further research focused on these molecules, mechanisms, or diseases may be gradually discovered, which may also lead to a second spurt of research.

### 4.4 Limitations

(1) We completed the retrieved work on 23 January 2024. Therefore, some of the latest published literature with key findings may be neglected in this study (Zhu et al., 2021, 2022). (2) We only retrieved articles and reviews from WoSCC because we wanted to guarantee the quality of the literature as much as possible. Therefore, other publication types, like books, case reports, clinical trials, and meta-analyses with important findings, may also be neglected (Peng et al., 2022; Xavier-Santos et al., 2022). (3) Because the search was limited to the WoSCC-indexed journals, a few studies were not included in the WoSCC (Zhu et al., 2022; Gu et al., 2023). (4) Only English studies were retrieved from the WoSCC, so those published in other languages with important research results may not be retrieved and concluded (Peng et al., 2022; Xavier-Santos et al., 2022). (5) Due to software limitations, bibliometric studies are analysed and created by mathematical methods based on machine-driven understanding (Lam et al., 2022; Wang et al., 2022). Therefore, the literature may have included irrelevant documents. Thus, manual and detailed screening and review of the analysed results are needed. These limitations have been reported in other bibliometric studies.

## 5 Conclusion

This study aimed to analyse research hotspots and trends in PTEN research in the nervous system, particularly in neural development and neurological diseases. Furthermore, a series of key regulated signaling molecules and cellular mechanisms related to PTEN in the nervous system have been identified, which could provide therapeutic targets and guidelines for PTEN-related nervous system diseases in fundamental research and clinical treatments.

## Data availability statement

The original contributions presented in the study are included in the article/supplementary material, further inquiries can be directed to the corresponding author.

## Author contributions

YZ: Investigation, Methodology, Project administration, Software, Writing – original draft. Y-tT: Formal analysis, Investigation, Methodology, Writing – original draft. M-jW: Investigation, Methodology, Software, Writing – original draft. LL: Data curation, Funding acquisition, Investigation, Methodology, Project administration, Software, Writing – review & editing. J-fH: Project administration, Supervision, Writing – review & editing. S-cW: Conceptualization, Formal analysis, Funding acquisition, Methodology, Project administration, Supervision, Writing – review & editing.

## Footnotes

^1^ https://www.NCBI.nlm.nih.gov/mesh
